# A therapeutic guide on pediatric irritable bowel syndrome and functional abdominal pain-not otherwise specified

**DOI:** 10.1007/s00431-022-04459-y

**Published:** 2022-04-23

**Authors:** Robyn Rexwinkel, Arine M. Vlieger, Miguel Saps, Merit M. Tabbers, Marc A. Benninga

**Affiliations:** 1 Emma Children’s Hospital, Amsterdam UMC, Pediatric Gastroenterology, University of Amsterdam, Room C2-312, PO Box 22700, 1100 DD Amsterdam, Netherlands; 2 Department of Pediatrics, St. Antonius Hospital, Nieuwegein, Netherlands; 3 Department of Pediatric Gastroenterology, University of Miami, Miami, FL USA

**Keywords:** Children, Management, Therapy, Treatment, Chronic abdominal pain

## Abstract

Disorders of the gut-brain interaction negatively impact quality of life and carry a substantial socioeconomic burden. Irritable bowel syndrome (IBS) and functional abdominal pain-not otherwise specified (FAP-NOS) are common functional abdominal pain disorders in childhood. The pathophysiology is not fully understood, and high-quality intervention trials and international guidelines are missing. Therefore, the management of these disorders remains challenging. This review aims to provide an up-to-date overview of therapeutic possibilities for pediatric IBS or FAP-NOS and recommends management strategies. To prevent unnecessary referrals and extensive costs, it is fundamental to make a positive diagnosis of IBS or FAP-NOS in children with chronic abdominal pain with only minimal investigations. A tailor-made approach for each patient, based on the accompanying physical and psychological symptoms, is proposed to date.

*Conclusion*: Shared decision-making including non-pharmacological and pharmacological interventions should be considered and discussed with the family.

**Table:** 

**What is Known:** *• Irritable bowel syndrome and functional abdominal pain-not otherwise specified are common in childhood.* *• Although the number of treatment options has grown recently, managing these disorders can be challenging and unsatisfactory, and no evidence-based international management guidelines are available.*	
**What is New:** *• We suggest using a stepwise individualized approach to management, where after first-line management, both non-pharmacological and pharmacological interventions should be discussed.*

**Supplementary information:**

The online version contains supplementary material available at 10.1007/s00431-022-04459-y.

## Introduction

Functional abdominal pain disorders (FAPDs) are disorders of the gut-brain axis characterized by chronic abdominal pain and altered bowel movements in the case of irritable bowel syndrome (IBS). FAPDs comprise four disorders: functional dyspepsia, IBS, abdominal migraine, and functional abdominal pain-not otherwise specified (FAP-NOS) (Supplemental Table 1) [1, 2]. In IBS, four types can be distinguished: predominant-diarrhea (IBS-D), predominant-constipation (IBS-C), mixed or alternating stool forms (IBS-A), and unclassified (IBS-U) [3].

FAPDs are common, with an estimated prevalence ranging from 1.6 to 41.2% in the pediatric population. These disorders have a profoundly negative impact on quality of life and carry a substantial socioeconomic burden [4–6]. Despite their high prevalence and impact, the pathophysiology underlying FAPDs is not well understood. FAPDs are presumably multifactorial and include genetic factors; psychological factors such as child abuse, stress, or depression; hypersensitivity to food products; and gut microbiota alterations [7]. Although the number of treatment options has grown recently, managing these disorders can be challenging and unsatisfactory. In this review, we aim to provide an up-to-date overview of therapeutic approaches, and we recommend management strategies focusing on pediatric IBS and FAP-NOS.

## Methods

We searched for relevant articles in English up to August 2021 in PubMed, MEDLINE, EMBASE, PsycINFO, and Cochrane Library. To identify unpublished or ongoing studies, the ClinicalTrials.gov register, the Current Controlled Trials meta-Register of Controlled Trials–active registers, and the WHO International Clinical Trials Registry Platform Search Portal were searched. To identify relevant articles and reviews missed by the search strategies, the reference lists from reviewed articles were searched by hand. Only randomized controlled trials and systematic reviews were included. The full search strategies are available upon request. Inclusion and exclusion criteria are presented in Supplementary File 1. We assessed the risk of bias of all included studies in earlier studies, using the Cochrane risk of bias tool [8–12].

## Management of IBS and FAP-NOS

Treatment often includes one or more of these strategies: (1) first-line management consisting of validation, explanation, and a positive diagnosis, (2) non-pharmacological treatment, and (3) pharmacological treatment.

1. First-line management
  The cornerstone of helping a child with IBS or FAP-NOS is first to validate the symptoms followed by a proper explanation of the diagnosis according to the biopsychosocial model [7]. An evidence-based, multidisciplinary treatment plan is essential to improve recovery and long-term prognosis [13].
  Validation, explanation, and a positive diagnosis. One of the first steps is to acknowledge that the pain is real even though no severe organ damage is present. It can be helpful to explain that the pain is caused by hypersensitive nerves, using metaphors like a fire alarm that keeps on alarming although there is no fire [13]. Enough time must be allocated to make a positive diagnosis by discussing all the evidence that supports your diagnosis of IBS or FAP-NOS. Education on the interplay of different biopsychosocial factors that generate and maintain chronic abdominal complaints is also helpful. Finally, one needs to elicit expectations and elucidate that the long-term prognosis is favorable. The primary treatment goal should not be the complete eradication of pain but optimization of daily functioning, including school participation, a normal sleep pattern, and participation in extracurricular activities [14, 15]. The practitioner should remain connected with patients and parents through email and/or phone contact and follow-up visits tailored to each case every 4–12 weeks to increase treatment adherence and reduce the feeling that patients and families are discharged and left without support.
  The parental response to their child’s abdominal pain. A multidisciplinary family approach is an essential part of the treatment strategy. An RCT studied the effects of parental attention versus distraction versus no instruction in children with chronic FAP [16]. Abdominal complaints were reduced by half in the distraction group and nearly doubled in the attention group. The study suggests that parental distraction is a powerful coping strategy. Moreover, Lindley et al. showed that healthcare consumerism in families lacking insight into their child’s problem can be harmful to the child with FAP [17]. Prognostic indicators of “healthcare consumerism” were refusal to engage with psychological services, involvement of more than three consultants, lodging of a manipulative complaint with hospital management by the child’s family, and lack of development of insight into psychosocial influences on symptoms [17].
  Identify psychological and physical stressors that may play a crucial role in a child’s abdominal pain experience and, possibly, help reverse them. Parental acceptance of the biopsychosocial model of illness has shown to be an important factor for symptom relief in children with FAPDs [18].
  Additional analgesic therapysuch as non-steroidal anti-inflammatory drugs, acetaminophen, and aspirin is sometimes used by general practitioners to treat pain. However, the efficacy of these drugs in treating pediatric chronic abdominal pain is not supported in any clinical trial and should be used with caution in clinical practice [19–21].
2. Non-pharmacological treatment

## Dietary interventions

In the last decade, there has been a great interest in the role of diet in the pathogenesis and management of FAPDs. More than 90% of children with a FAPD report that at least one food is associated with deterioration of their GI symptoms. As a result, children frequently avoid foods and implement diet strategies [22, 23]. However, it is likely that these food-associated symptoms are more the result of the gastrocolic reflex than that they are caused by food intolerances [24–26]. Indeed, research has shown little evidence that dietary interventions are helpful for this population [15, 27, 28]. There is some evidence regarding probiotics and dietary fibers, such as psyllium fibers [9, 12]. A detrimental effect of gluten is frequently self-reported [29]. Non-celiac gluten sensitivity is a clinical condition that has been insufficiently studied in children but may contribute to trigger or worsen GI symptoms (Table 1) [30].

**Table 1:** Non-pharmacological interventions

Intervention	Participants	Results
*Fibers*		
Psyllium [35]^a^	Children 7–18 years (*N* = 103) IBS (Rome II criteria)	Improvement in reduction of mean number of pain episodes (8.2 ± 1.2 vs 4.1 ± 1.3; *P* = 0.03); no difference in pain intensity
Soluble fiber [25]^a^	Children 4–18 years (*N* = 385) FAPD (Rome II, III, IV criteria)	Difference in treatment success in favor of soluble fiber group (RR 2.40, 95% CI 1.10–5.25; NNT = 3, 4 studies, 268 participants); no difference in pain intensity after soluble fiber treatment (SMD—0.63, 95% CI − 1.61 to 0.35; 2 studies, 135 participants)
*Low FODMAP diet*		
Low FODMAP diet [46]^b^	Children 5 to 12 years (*N* = 29) FAP (Rome III) FAP-NOS (Rome IV)	No significant differences apparent in pain frequency and intensity between the two diets
Low FODMAP diet [47]^c^	Children 7 to 17 years (*N* = 33) IBS (Rome III criteria)	Treatment success defined as ≥ 50% decrease in frequency of abdominal pain episodes (50% vs 59%; *P* > 0.05); significant improvement in abdominal pain episodes/day (1.1 ± 0.2 vs 1.7 ± 0.4; *P* < 0.05)
*Probiotics*		
*Lactobacillus reuteri* DSM [24]^a^	Children 4–18 years (*N* = 360) IBS/FAP (Rome III criteria)	Difference in treatment success in favor of *Lactobacillus reuteri* group (RR 1.33, 95% CI 0.86 to 2.4; 5 studies, 178 participants); difference in complete resolution of pain in favor of *Lactobacillus reuteri* group (RR 1.35, 95% CI 0.76 to 2.41; 4 studies, 151 participants); difference in frequency of pain (episodes/week) in favor of *Lactobacillus reuteri* group (RR − 0.14, 95% CI − 1.18 to 0.90; 3 studies, 116 participants)
*Lactobacillus Rhamnosus* CG [24]^a^	Children 5–16 years (*N* = 245) IBS/FAP (Rome II criteria)	Difference in treatment success in favor of *Lactobacillus rhamnosus CG* group (RR 1.57, 95% CI 0.73 to 3.34; 2 studies, 123 participants); difference in complete resolution of pain in favor of *Lactobacillus rhamnosus CG* group (RR 2.60, 95% CI 1.00 to 6.77; 1 study, 52 participants); difference in frequency of pain (episodes/week) in favor of *Lactobacillus rhamnosus CG* group (RR − 0.57, 95% CI −0.81 to −0.33; 2 studies, 122 participants)

*AP-FGIDs* abdominal pain predominant functional gastrointestinal disorders, *CAP* chronic abdominal pain, *FAP* functional abdominal pain, *FD* functional dyspepsia, *FGID* functional gastrointestinal disorder, *FODMAP* fermentable oligosaccharides, disaccharides, monosaccharides, and polyols, *IBS* irritable bowel syndrome, *IBS-C* irritable bowel syndrome, predominant constipation, *NICE* National Institute for health and Care Excellence, *RAP* recurrent abdominal pain

^a^Compared with placebo

^b^compared with diet based on the NICE guidelines

^c^compared with American diet

### Dietary fiber

A normal fiber intake is recommended for every child [31, 32]. Inadequate fiber intake has been proposed as a risk factor for developing FAPDs in children [33, 34]. Increasing dietary fiber intake was recommended as first-line treatment for IBS since fibers potentially decrease intracolonic pressure, accelerate gut transit time, and reduce abdominal pain [35, 36]. Soluble fibers may be particularly useful in the management of IBS-C, since they attract water into stools and therefore may relieve symptoms of constipation [37, 38]. However, increased gas production may also occur due to fiber fermentation [39]. A meta-analysis of adult studies has shown the benefit of soluble fibers, such as psyllium, as opposed to insoluble fiber, such as bran [40, 41]. Therefore, adult IBS clinical guidelines support soluble fiber in IBS treatment [39, 42]. A recent meta-analyses in children with FAPDs, including five RCTs, found some beneficial effects for the use of soluble fibers, in particular psyllium, with a number needed to treat of 3. Certainty of the evidence is very low, but given the low cost, absence of serious side effects, and easy availability, soluble fiber may be considered in daily practice [9].

### Low FODMAP diet

Studies in adults have shown the beneficial effect of a diet low in fermentable oligosaccharides, disaccharides, monosaccharides, and polyols (FODMAP) for the treatment of IBS [43]. It is hypothesized that one of the mechanisms of action involves a reduction in gas production and subsequently in luminal distention, resulting in a decrease in pain [43, 44]. A meta-analysis of adult studies on the efficacy of the low FODMAP diet showed a reduction in GI symptoms and an improved quality of life [45]. However, adherence to the low FODMAP diet is difficult, it involves high cost, and the involvement of a dietician is essential to achieve nutritional adequacy and successful treatment outcomes [46–48]. It is unknown when and how eliminated foods should be reintroduced, but continuing a low FODMAP diet for longer than 6 weeks is accompanied with the risk of malnutrition [49, 50]. To date, evidence-based recommendations to support the use of the low FODMAP diet in the pediatric population are lacking. Only two low-quality RCTs have been conducted, showing no efficacy, but more data from well-designed studies are needed before definitive conclusions can be drawn [51, 52]. To make the low FODMAP diet more available, new methods need to be implemented in clinical practice. The use of online apps and the widespread use of dietician-led groups may play an important role in near future [53, 54].

### Gluten-free diet

In the last decade, adult studies have highlighted the potential role of gluten sensitivity as a trigger of GI symptoms in IBS [29, 30, 55]. This condition is known as non-celiac gluten sensitivity. IBS patients frequently report gluten sensitivity in the absence of a celiac disease diagnosis [30]. Future research is required to investigate the role of non-celiac gluten sensitivity in children with IBS. Currently, two pediatric IBS trials are underway, one being a double-blind placebo-controlled crossover trial evaluating the prevalence of gluten sensitivity (NCT02431585) and the other evaluating a gluten-free diet compared to a low FODMAP diet (NCT03694223).

### Probiotics

Probiotics are defined as “live microorganisms that, when administered in adequate amounts, confer a health benefit on the host” [56]. Probiotics are used to restore the altered microbiome composition, hamper the overgrowth of potentially pathogenic bacteria, and alter intestinal inflammation and permeability [57–59]. Since there is growing evidence for the role of the microbiome in the pathogenesis of FAPDs, probiotics may be a promising treatment option [60, 61]. A recently published Cochrane review evaluated the efficacy and safety of probiotics in children with FAPDs [12]. Meta-analyses showed moderate to high-quality evidence for the effectiveness of *Lactobacillus rhamnosus* GG and *Lactobacillus reuteri* DSM in successfully treating IBS and FAP in children [12]. There is limited evidence for the use of VSL#3.

### Psychological interventions

Psychosocial interventions, such as cognitive behavioral therapy (CBT) and hypnotherapy (HT), have proven to be successful in the management of pediatric FAPDs (Table 2) [15].

**Table 2:** Psychological interventions

Intervention	Participants	Results
*Cognitive Behavioral Therapy*		
CBT vs no intervention [65]	Children 6–18 years (*N* = 785) FAPDs (Rome II,III, IV criteria) and RAP (Apley criteria)	Difference in treatment success in favor of CBT group (38% vs 15%) (RR 2.37, 95% CI 1.30 to 4.34; NNT = 5, 6 studies, 324 participants) CBT leads to lower pain frequency (RR − 0.36, 95% CI 0.63 to − 0.09; 7 studies, 446 participants) CBT leads to lower pain intensity (RR − 0.58, 95% CI 0.83 to − 0.32; 6 studies, 332 participants)
CBT vs educational support [65]	Children 5–18 years (*N* = 975) FAPDs (Rome III, IV criteria) and RAP (Apley criteria)	No difference in pain intensity between CBT group and educational support group (MD − 0.36, 95% CI 0.87 to − 0.15; 1 study, 127 participants) No difference in composite pain scores (MD − 0.07, 95% CI − 0.29 to 0.15; 1 study, 300 participants)
*Hypnotherapy and guided imagery*		
HT vs no intervention [65]	Children 6 to 18 years (*N* = 91) IBS/FAP (Rome II, III)	Difference in treatment success in favor of HT group (56% vs 19%) (RR 2.86, 95% CI 1.19 to 6.83; NNT = 5, 2 studies, 91 participants)
Gut-directed HT vs HT [65]	Children 6 to 17 years (*N* = 73) IBS/FAP (Rome III criteria)	In both groups, results suggest a high efficacy of standardized home-based HT
Audio-recorded guided imagery vs no intervention [72]	Children 6 to 15 years (*N* = 34) FAP (Rome II)	ITT-analysis, significant difference in treatment responders (63% vs 27%; P = 0.03; NNT = 3);
Home-based HT vs iHT [71]	Children 12 to 18 years (*N* = 260) IBS/FAP (Rome III criteria)	Home-based HT by using a CD was non-inferior to individual HT group (62.1% vs 71%; *P* = 0.002) at 1-year follow-up
Yoga		
Yoga vs no intervention [65]	Children 8 to 18 years (*N* = 127) IBS/FAP (Rome I, III criteria)	No difference in treatment success between both groups (28% vs 24%; *P* = 0.78) (RR 1.09, 95% CI 0.58 to 2.08, 2 studies, 99 participants)
Neurostimulation		
Electrical neurostimulation (PENFS) [84]^a^	Children 11 to 18 years (*N* = 115) AP-FGIDs (Rome III criteria)	Significant difference in lower median PFSD composite scores with a mean decrease of 11.48 (95% CI 6.63 to 16.32; *P* < 0·0001); lower worst pain scores (*p* < 0·0001); improved global well-being (*p* = 0·0003) after 3 weeks; greater reduction in median PFSD composite scores (*p* = 0·018), and worst pain (*p* < 0·0001) compared with sham at long-term follow-up

*AP-FGID* abdominal pain-related functional gastrointestinal disorder, *CBT* cognitive behavioral therapy, *FAP* functional abdominal pain, *FAPD* functional abdominal pain disorder, *FGID* functional gastrointestinal disorder, *HT* hypnotherapy, *IBS* irritable bowel syndrome, *NNT* number needed to treat, *RAP* recurrent abdominal pain, *PENFS* percutaneous electrical nerve field stimulation

^a^Compared with sham

### Cognitive behavior therapy

Cognitive behavioral therapy (CBT) aims to alter the behaviors, cognitions, and emotions, that may contribute to IBS symptom escalation or maintenance [62–64]. Children and parents are taught to implement different coping and distraction strategies, and often also relaxation techniques, to decrease symptoms. CBT can be provided in various settings, such as face-to-face therapy [65–67], to parents via the telephone, [68] or targeted to children via the Internet [69–71]. A systematic review and meta-analysis in children aged 4–18 years with FAPDs included 17 studies (*N* = 1760) of CBT [10]. This SR found moderate certainty evidence that CBT leads to significant reduction in pain intensity and frequency scores compared with no intervention with a number needed to treat of 5. There was low certainty evidence that found that there is no difference between CBT and educational support in reducing pain intensity and frequency scores. Limitations of CBT are that there may be limited access to mental health professionals and that insurance may not cover treatment. To overcome the low availability of mental health professions, Internet-delivered and telephone-delivered CBT have shown to be effective alternatives, potentially reduce healthcare costs, and increase the availability of treatment [69–72].

### Hypnotherapy

In HT, a patient is induced into a hypnotic state. During this state, a therapist guides the patient to respond to suggestions to alter its subjective experiences, perception, emotion, sensation, and thoughts or behavior [73, 74]. HT can be provided individually by a therapist [75, 76], or home-based by the use of HT-exercises on CD [77, 78]. Eight RCTs of children with IBS or FAP-NOS (6–18 years of age; *N* = 496) found low certainty results indicating that HT (both individually by a therapist or as self-exercise using a CD) may be an effective treatment option (number needed to treat = 5) [10]. Even in the long-term, there is a continued benefit of HT at 5-years follow-up [79, 80]. One of the disadvantages of HT is the lack of enough well-trained hypnotherapists, its time investment, and the lack of coverage by healthcare insurances. Home-based HT using standardized scripts is an attractive alternative treatment option and was originally developed to make hypnosis for children with IBS and FAP-NOS more widely available, especially in countries or areas with a low number of licensed hypnotherapists or with high costs for therapist. It has proven to be non-inferior to individual HT by a therapist at 1-year and 5-year follow-up [77, 80]. To date, online packages with ready-to-use HT exercises for at home use, together with an instruction manual and additional video material, are available for children in English, Spanish, and Dutch [81–83].

### Yoga

Yoga practice using meditation techniques and breathing practices in combination with physical poses has been shown to improve body tone, reduce anxiety, and heighten feelings of well-being [84]. Three RCTs, including 127 children with IBS or FAP, have been performed to evaluate the effect of yoga [85–87]. After meta-analysis, no differences in treatment success were found between the yoga intervention and the control group [10]. Studies were of low quality since only small groups of children were included and methodological shortcomings. Therefore, there is no evidence to recommend yoga as a routine intervention in the management of pediatric FAPDs.

### Other forms of complementary and alternative medicine

To date, the efficacy of complementary therapies such as acupuncture, herbal therapy, homeopathy, chiropractic therapy, or osteopathy have not been evaluated in pediatric clinical FAPD trials [10]. However, these alternative therapies are used by about 40% of children diagnosed with FAPDs [88, 89]. Potential reasons for using complementary and alternative medicine are the lack of perceived benefit of conventional therapy and its associated side effects [89]. More research in this field is clearly needed.

### Other treatments

#### Neurostimulation

Percutaneous electrical nerve field stimulation (PENFS) to the outer ear targets specific pain areas in the central nervous system. By stimulating auricular branches of nerves that allow accessing the central nervous system, also visceral hypersensitivity can be modulated [90]. A large randomized, sham-controlled study assessed the efficacy of PENFS in the external ear in 115 children with FAPDs. Compared with the sham control group, PENFS treatment improved well-being with a significant reduction in pain and disability. Furthermore, beneficial effects were sustained at follow-up [90]. Although more evidence is needed, these data suggest that PENFS may be a good and safe non-pharmacological treatment option for pediatric FAPDs.

### Fecal microbiota transplantation

Fecal microbiota transplantation targets the microbiome and may be a potential future therapeutic strategy in IBS patients. However, results in adult IBS studies have shown conflicting results and data in the pediatric population is lacking. Therefore, no valid conclusions on the efficacy of this treatment for pediatric IBS can be drawn [91, 92]. Currently, an RCT is assessing the use of fecal microbiota transplantation for refractory IBS in adolescents (NCT03074227).

## Pharmacological treatment

Based on the current evidence, it is not possible to recommend any specific pharmacological treatment for the treatment of pediatric FAPDs [11]. The efficacy of several agents has been assessed for the treatment of pediatric FAPDs. Information on these studies is shown in Table 3.

**Table 3:** Pharmacological interventions

Intervention	Participants	Results
*Antispasmodics*		
Peppermint oil [91]^a^	Children 4–13 years (*N* = 120) FGIDs (Rome III criteria)	Compared with placebo a decrease in pain severity (*P* = 0.001), pain duration (*P* = 0.0001) and pain frequency (*P* = 0.0001)
Trimebutine [95]^a^	Children 4–18 years (*N* = 78) IBS (Rome III criteria)	Overall clinical recovery (in pain or discomfort) (95% vs 21%; *P* < 0.0001)
Peppermint oil [92]^a^	Children 8–17 years (*N* = 42) IBS (Rome I/Manning criteria)	Treatment success (pain severity) defined as “better” or “much better” (71% vs 43%; *P* < 0.001); significantly decrease in pain intensity in peppermint oil group (*P* < 0.03)
Drotaverine [93]^a^	Children 4–12 years (*N* = 132) RAP (Apley criteria)	Significant reduction of pain episodes (*P* = 0.01); decrease in school absenteeism (*P* = 0.05)
Mebeverine [94]^a^	Children 6–18 years (*N* = 115) FAP (Rome III criteria)	Response rate defined as reduction in pain (41% vs 30%; *P* = 0.117)
*Antidepressants*		
Citalopram [99]^a^	Children 6–18 years (*N* = 115) FAP (Rome III criteria)	Responded (pain) to treatment at 4 weeks (41% vs 30%; *P* = 0.17); responded (pain) to treatment at 8 weeks (53% vs 41%; *P* = 0.15)
Amitriptyline [100]^a^	Children 8–17 years (*N* = 90) FAP, FD, IBS (Rome II criteria)	Satisfactory relief (59% vs 52%; *P* = 0.81); no significant difference in pain intensity scores; large placebo response reported
Amitriptyline [101]^a^	Children 12 to 18 years (*N* = 33) IBS (Rome II criteria)	Improvement in overall quality of life (39% vs 0%; *P* = 0.013); improvement in periumbilical pain at week 10 (*P* = 0.018); no significant differences in pain frequency and intensity
*Antibiotics*		
Rifaximin [111]^a^	Children 8–18 years (*N* = 75) CAP (Rome II criteria)	No significant differences apparent in pain frequency and intensity between both groups
Rifaximim [110]^c^	Children 3–15 years (*N* = 50) IBS (Rome II criteria)	Benefit in improving abdominal pain, bloating, and flatulence (*P* < 0.005)
*Prokinetics*		
Domperidone [123]^a^	Children 5–12 years (*N* = 100) AP-FGIDs (Rome III criteria)	Improved cure rate (44% vs 28%; *P* = 0.028), decreased severity of abdominal pain (54% vs 30%; *P* = 0.008)
*Laxatives*		
PEG 3350 + Tegaserod [113]^d^	Children 13 – 18 years (N = 48) IBS-C (Rome II criteria)	Significant improvement as a reduction in pain (67% vs 19%; *P* < .05); statistically significant different in pain intensity between the two groups in favor of the tegaserod group (*P* < .05)

*AP-FGIDs* abdominal pain predominant functional gastrointestinal disorders, *CAP* chronic abdominal pain, *FAP* functional abdominal pain, *FD* functional dyspepsia, *FGID* functional gastrointestinal disorder, *IBS* irritable bowel syndrome, *IBS-C* irritable bowel syndrome, predominant constipation, *RAP* recurrent abdominal pain

^a^Compared with placebo

^b^compared with usual care

^c^open-trial

^d^compared with PEG350

### Antispasmodics

Antispasmodic agents act directly on the intestinal smooth muscles to ensure relaxation, or indirectly on the nerves of the intestinal smooth muscles via receptor blockade, decreasing gastrointestinal contractions, and, consequently, alleviating abdominal pain complaints [93–95]. Only five RCTs have been conducted on the use of antispasmodics in children. Two studies investigated the effect of peppermint oil [96, 97], and three investigated drotaverine [98], mebeverine [99], or trimebutine [100]. A recent meta-analysis found a significant difference in treatment success between the antispasmodic and placebo groups. No difference was found in withdrawals due to adverse events [11]. However, the overall quality of the studies was very low, and results should therefore be interpreted with caution. Furthermore, these RCTs comprise small sample sizes, short-duration of therapy, and limited follow-up. More data are needed before definitive conclusions can be drawn. Currently, an RCT is investigating the effectiveness of mebeverine on abdominal pain reduction in children with IBS or FAP-NOS (Trial NL7508).

### Antidepressants

Antidepressants, such as amitriptyline and citalopram, are central neuromodulators affecting the brain-gut axis. They have anticholinergic effects, decrease visceral sensitivity and GI motility, and improve mood and sleep patterns [101, 102]. A recent Cochrane review, including three RCTs, found insufficient evidence to support the use of antidepressants (amitriptyline and citalopram) in children with FAPDs [103–106]. Currently, antidepressants are commonly used in clinical practice for children who do not respond to first-line treatments [107]. However, some safety issues regarding these agents should be considered. In 2004, the Food and Drug Administration (FDA) issued boxed warnings on antidepressant drugs due to a potential increased risk of suicidality in the pediatric population [108]. In addition, the practitioner should be cautioned of the potential risk of cardiac-related side effects of tricyclic antidepressants (TCAs). Current practice advises performing an electrocardiogram to screen for prolonged QT intervals or bundle branch block before the administration of TCAs and advising families about the risks [109]. However, studies found no correlation between serious adverse cardiac events and the use of low-dose TCA in pediatric FGIDs, and side effect risks are usually reduced over time [110, 111]. More research is needed to draw firm conclusions.

### Antibiotics

Rifaximin is a nonabsorbed antibiotic, which is thought to eliminate small-intestinal bacterial overgrowth. Since it is hypothesized that IBS-D patients have an abnormal microbiome, rifaximin may be a potential treatment for GI disorders [112–114]. In adult IBS, the use of rifaximin to treat global IBS-D symptoms has shown to be effective and safe [42, 115]. In the pediatric population, two trials were conducted on the efficacy of rifaximin. The first trial showed that, in 50 children with IBS and an abnormal lactulose breath hydrogen test, rifaximin significantly improved abdominal pain, bloating, and flatulence [116], while the other RCT, evaluating rifaximin in 75 children with FAP, found no significant difference in pain scores [117]. To date, rifaximin in pediatric IBS is not recommended. There is a long-term safety concern of rifaximin use as it may produce cross-resistant bacterial strains and interfere with the healthy microbiome in children [118].

### Laxatives

A small study investigated polyethylene glycol 3350 (PEG) and tegaserod in children with IBS-C. The study found significant improvements in pain scores in the PEG + tegaserod treatment group compared with the PEG-alone group [119]. No evidence exists that PEG reduces abdominal pain in patients with IBS-C. However, PEG is commonly used as a first-line treatment for constipation since it is effective and safe. Therefore, it could be recommended to treat symptomatic constipation in IBS-C.

The relatively new therapeutic agents prucalopride (a 5-HT_4_ receptor agonist), and lubiprostone (prostaglandin E_1_ derivative) and linaclotide and plecanatide (both a guanylyl cyclase agonist) (both licensed for the management of IBS-C in adults) have shown benefits in adults with IBS-C [120–122]. Neither of these agents have proven efficacy in the pediatric population and are currently not approved for the treatment of IBS-C in children. Lubiprostone has been studied only in children with functional constipation and showed conflicting results [123–125]. Recently, the efficacy and safety of different dosages of linaclotide were evaluated in a phase 2 trial for IBS-C in children, with limited but promising results (NCT02559817). Thus, there is a clear need for large placebo-controlled RCTs evaluating the efficacy of this new compound in children with IBS-C before making any recommendations for its use.

### Prokinetics

Dopamine antagonists, such as domperidone, have beneficial effects in adults with functional dyspepsia and IBS [126–129]. Only a single placebo-controlled trial assessed the efficacy of domperidone in children with FAPDs (*n* = 100) [130]. There was no significant difference in treatment success after 8 weeks of treatment. However, there was a significant decrease in abdominal pain intensity in the domperidone group compared with placebo. No side effects were reported. Children with FAPDs often report other symptoms, such as nausea, which is experienced by about half of the children at least twice a week [131, 132]. Therefore, domperidone treatment can be used as symptomatic treatment in children with comorbid nausea. However, caution is warranted since the use of domperidone has been associated with prolonged QT intervals and is therefore not licensed in children under the age of 12 [133, 134].

### Antidiarrheal agents

Loperamide is an over-the-counter opioid receptor agonist commonly used in clinical practice to treat diarrhea [135–137]. However, guidelines do not recommend it as first-line treatment for adults with IBS-D since it is not effective for the most bothersome IBS symptoms, abdominal pain, and bloating [121, 135]. Although no RCTs have evaluated the efficacy of loperamide in children with IBS-D, it may still be considered for the symptomatic treatment in children with IBS-D [136].

### Bile acid sequestrants

In adult and pediatric patients with IBS-D, there is some evidence that a subset of these patients has bile acid malabsorption [138–140]. This suggests that bile acid sequestrants could play a role in treating diarrheal symptoms in IBS. Several agents have indeed been shown to improve stool consistency in adults with IBS-D, such as cholestyramine, colestipol, and colesevelam [138, 141, 142]. To date, no well-designed studies have evaluated their efficacy in children with IBS.

### Placebo

In pediatric FAPDs, the placebo response is substantial, with on average 41% of children improving on placebo [143]. Different factors are significant contributors to the placebo effect, such as the natural course of the disease, methodological bias, regression to the mean, and contextual factors. Contextual factors, including expectations and conditioning are known as the “true placebo-effect” [144–146]. Healthcare professionals should be mindful of the “true placebo-effect,” since this can be influenced by an active listening approach and a warm physician–patient relationship, potentially leading to positive patient expectations and thus improved treatment responses [147, 148].

It is interesting to better understand whether the placebo-effect is still present when the patient is aware of taking a placebo. A study in children has shown a beneficial effect of non-deceptive placebo in children with FAPDs [149]. A large open-label study is currently assessing the efficacy of open-label placebo in children with FGIDs (NCT02389998). Similar trials in adults with IBS have shown promising results [150, 151].

### Novel treatments in adults with IBS

In the adult population, management is mostly based on the predominant symptom of the bowel dysfunction: constipation/bloating (IBS-C) or diarrhea (IBS-D) [152].

In adults with constipation-predominant IBS several treatment are in development [153]. Mizagliflozin (a SGLT1 inhibitor) reduces the uptake of sodium ions from the lumen, resulting in water retention in the lumen and loose stools. A phase 2 placebo-controlled trial in adults with IBS-C showed that mizagliflozin had significantly higher response rates than placebo and also appeared to be safe [154]. Furthermore, tenapanor (a sodium-hydrogen exchanger inhibitor) have proven to be effective and safe in phase 2 trials in adults with IBS-C [155].

Novel approaches for adults with IBS-D include opioid mediators, such as eluxadoline (a mixed opioid receptor agonist and antagonist), which has shown to be effective and safe [115, 156, 157]. However, eluxadoline has limitations in its use, since patients with a previous cholecystectomy report sphincter of Oddi spasms and pancreatitis [157]. The efficacy of eluxadoline is currently assessed in adolescents with IBS-D (NCT03339128).

Serum bovine-derive immunoglobulin (SBI) modulates junctional regulatory proteins in the gut and may therefore be a potential effective treatment [158]. Two pilot RCTs in adolescents with IBS-D have examined the effect of this drug, but have shown conflicting results [159, 160].

Ibodutant is a selective neurokinin-2 receptor antagonist and has proven to be effective and safe in phase 2 trials in adults with IBS-D [161].

## Putting it all together

**Fig. 1:**
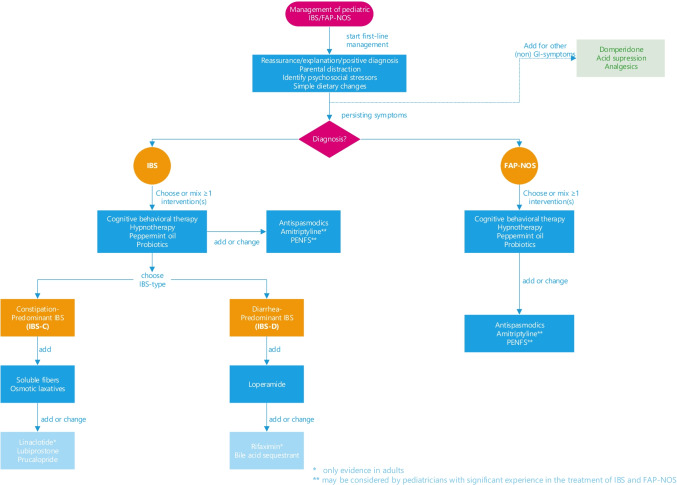
Flow diagram of treatment

The heterogeneity of pediatric IBS and FAP-NOS, even within individual subtypes, makes it challenging to design a treatment algorithm to fit all children. It is known that up to 40% of children remain symptomatic despite treatment [162–164]. A stepwise approach, including a positive diagnostic strategy with minimal investigations, involving patients and families in shared decision-making, and an individualized approach to management, is the fundaments of IBS and FAP-NOS management. We propose a tailor-made approach for each patient, based on the family’s beliefs, published evidence when available, and the treatment of comorbid symptoms such as nausea, bloating, diarrhea, or constipation. Both non-pharmacological and pharmacological interventions should be discussed (Fig. 1). The first recommended step in the management of both IBS and FAP-NOS is validation, education, providing a positive diagnosis, and identifying stress factors. Initial treatment should include parental distraction, and simple dietary changes. When symptoms persist, especially in patients with functional disability, (online) psychological treatments could be proposed since those have proven to be successful therapies. However, while CBT or HT might be accepted by some, others might prefer pharmacological therapies or a combination of interventions. It is important to emphasize that although there is limited data to substantiate the efficacy of the combination of different interventions, those could be combined. If patients have IBS with constipation, we recommend increasing soluble fibers or laxatives, such as PEG. Diarrhea may be ameliorated with loperamide. For children with troublesome and persistent IBS-D symptoms, rifaximin and bile acid sequestrants may help. Special attention should be paid to non-abdominal pain symptoms, such as headache and chest-, back-, joint-, and extremity (arms and legs) pain [165]. These comorbid somatic symptoms are present in almost 75% of children and are associated with increased abdominal pain frequency and severity, functional disability, poor sleep, psychosocial distress, and lower health-related quality of life, potentially influencing long-term prognosis [165–168]. Additional therapy with analgesics, such as non-steroidal anti-inflammatory drugs or paracetamol, could be considered to treat these complaints. It is important to emphasize that the majority of patients can be treated with first-line management. However, various highly effective therapies (dietary and psychological interventions) are not easily available as a result of a lack of insurance coverage and also because of a lack of allied healthcare professionals. New developments include the delivery of online psychological therapies, through audiotapes, by phone, or via the Internet. Referral to a pediatric gastroenterologist experienced in pain disorders is required if first-line management fails, or if therapy to TCAs, such as amitriptyline, and PENF is considered, since these treatments are not commonly used in daily clinical practice.

A multidisciplinary approach to provide patient support is ideal, however, not always possible.

## Conclusion

In conclusion, IBS and FAP-NOS are common in childhood, though no evidence-based international management guidelines are available. We suggest using a stepwise individualized approach to management, where after first-line management, both non-pharmacological and pharmacological interventions should be discussed. More high-quality intervention studies in these patient groups are necessary to guide adequate clinical management in the future.

## Supplementary information

Below is the link to the electronic supplementary material.

Supplementary file1 (DOCX 20 KB)

## Abbreviations

CBT: Cognitive behavior therapy
FAP: Functional abdominal pain
FAPD: Functional abdominal pain disorders
FAP-NOS: Functional abdominal pain-not otherwise specified
FGID: Functional gastrointestinal disorder
FODMAP: Fermentable oligosaccharides, disaccharides, monosaccharides, and polyols
HT: Hypnotherapy
IBS: Irritable bowel syndrome
IBS-C: Irritable bowel syndrome, predominant constipation
IBS-D: Irritable bowel syndrome, predominant-diarrhea
IBS-M: Irritable bowel syndrome mixed or alternating stool forms
IBS-U: Irritable bowel syndrome unclassified
GI: Gastrointestinal
PEG: Polyethylene glycol
PENFS: Percutaneous electrical nerve field stimulation
TCA: Tricyclic antidepressant
