# Supplementary material for: A therapeutic guide on pediatric irritable bowel syndrome and functional abdominal pain-not otherwise specified
Source: Eur J Pediatr. 2022 Apr 23;181(7):2603–17. doi: 10.1007/s00431-022-04459-y (PMC9192445; doi:10.1007/s00431-022-04459-y)
Supplement: Supplementary file 1 — Supplementary file1 (DOCX 20 KB) [file 431_2022_4459_MOESM1_ESM.docx]

| **Supplementary File 1. Eligibility criteria** |
| --- |
| **Inclusion criteria** |
| Study was a systematic review or RCT |
| Study population consisted of children aged 4 – 18 years |
| Functional abdominal pain disorders (FAPDs) were diagnosed, treated, or its course followed. FAPDs included:   - Irritable bowel syndrome (IBS); - Functional dyspepsia (FD); - Abdominal migraine (AM) and - Functional abdominal pain- not otherwise specified (FAP-NOS). |
| FAPDs in alignment with Rome criteria, other international criteria or a precise definition by the author |
| Dietary interventions were:   - FODMAP - (Additional) fiber intake - Lactulose- free diet - Gluten- free diet - Probiotics - Histamine- free diet - Decrease in gas producing foods - Extra fluid intake   Psychosocial and other interventions were:   - Behavior therapy: cognitive-behavioral therapy , hypnotherapy - Complementary and alternative medicine (acupuncture, homeopathy, body-oriented therapy, musculoskeletal therapy (osteopathy/chiropractic), yoga) - Fecal microbiota transplantation   Pharmacological interventions were:   - Antispasmodics - Antidepressants - Laxatives - Antidiarrheal agents - Antibiotics - Analgesics - Anti-reflux agents - Antiemetic agents - Antimigraine agents - Antihistaminic agents - Serotonergic agents |
| Outcomes measures* (assessed before and after start with treatment) were:   - Treatment success as defined by the authors (to be reported) - Pain frequency or change in frequency of pain - Pain intensity or change in pain intensity - Withdrawal due to adverse events - Quality of life or change in quality life measured using any validated defined measurement tool. - Anxiety/depression using any validated defined measurement tool. - Serious adverse events - Adverse events - Stool consistency *(disease-specific (IBS-C/D))* as defined by authors (Bristol stool or similar) at study end - Frequency of defecation at study end - Adequate relief (as reported by patient or parent) - School attendance or change in school attendance or performance |
| **Exclusion criteria** |
| Studies including children with:   - Hirschsprung’s disease; - Previous bowel surgery and - Complex congenital disorders. |
| If the range of children’s age was wider than 4-18 years, authors were requested for separate data of the children aged 4-18 years. If not available or no response the study was excluded; |
| Quasi randomized, none randomized, cohort, case control, animal studies, editorial, commentary, case reports; |
| Abstract were considered if they met the inclusion criteria, if not enough data to judge was presented, the authors were contacted and if no response was received within two weeks, abstracts were excluded. |
| If inclusion could not be decided based on full text, authors were contacted. If no response was received within two weeks, the study was excluded. |
| **outcome measures were identified according to the ‘çore outcome set’ for FAPDs* |
